# Sub-Emetic Toxicity of *Bacillus cereus* Toxin Cereulide on Cultured Human Enterocyte-Like Caco-2 Cells

**DOI:** 10.3390/toxins6082270

**Published:** 2014-08-04

**Authors:** Andreja Rajkovic, Charlotte Grootaert, Ana Butorac, Tatiana Cucu, Bruno De Meulenaer, John van Camp, Marc Bracke, Mieke Uyttendaele, Višnja Bačun-Družina, Mario Cindrić

**Affiliations:** 1 Laboratory of Food Microbiology and Food Preservation, Ghent University, Ghent B-9000, Belgium; E-Mail: Mieke.Uyttendaele@UGent.be; 2 Laboratory of Food Chemistry and Human Nutrition, Ghent University, Ghent B-9000, Belgium; E-Mails: Charlotte.Grootaert@UGent.be (C.G.); Tatiana.Cucu@UGent.be (T.C.); bruno.demeulenaer@UGent.be (B.D.M.); John.VanCamp@UGent.be (J.C.); 3 Laboratory for Biology and Microbial Genetics, Faculty of Food Technology and Biotechnology, Zagreb University, Zagreb HR-10000, Croatia; E-Mails: abutorac@pbf.hr (A.B.); visnjabd@pbf.hr (V.B.-D.); 4 Laboratory of Experimental Cancer Research, University Hospital Ghent, Ghent B-9000, Belgium; E-Mail: Marc1.Bracke@UGent.be; 5 Laboratory for System Biomedicine and Centre for Proteomics and Mass Spectrometry, “Ruđer Bošković” Institute, Zagreb HR-10000, Croatia; E-Mail: mcindric@irb.hr

**Keywords:** *Bacillus cereus*, cereulide, emetic toxin, doses, cell, toxicity, differentiated Caco-2

## Abstract

Cereulide (CER) intoxication occurs at relatively high doses of 8 µg/kg body weight. Recent research demonstrated a wide prevalence of low concentrations of CER in rice and pasta dishes. However, the impact of exposure to low doses of CER has not been studied before. In this research, we investigated the effect of low concentrations of CER on the behavior of intestinal cells using the Caco-2 cell line. The MTT (mitochondrial 3-(4,5-dimethylthiazol-2-yl)-2,5-diphenyltetrazolium bromide) and the SRB (sulforhodamine B) reactions were used to measure the mitochondrial activity and cellular protein content, respectively. Both assays showed that differentiated Caco-2 cells were sensitive to low concentrations of CER (in a MTT reaction of 1 ng/mL after three days of treatment; in an SRB reaction of 0.125 ng/mL after three days of treatment). Cell counts revealed that cells were released from the differentiated monolayer at 0.5 ng/mL of CER. Additionally, 0.5 and 2 ng/mL of CER increased the lactate presence in the cell culture medium. Proteomic data showed that CER at a concentration of 1 ng/mL led to a significant decrease in energy managing and H_2_O_2_ detoxification proteins and to an increase in cell death markers. This is amongst the first reports to describe the influence of sub-emetic concentrations of CER on a differentiated intestinal monolayer model showing that low doses may induce an altered enterocyte metabolism and membrane integrity.

## 1. Introduction

Cereulide (CER) is the main virulence factor of the emetic type of foodborne pathogen, *Bacillus cereus*. It is a heat-, protease- and pH-stable hydrophobic dodecadepsipeptide with a molecular mass of 1.2 kDa [1,2]. It comprises three repetitions of tetrapeptide motifs synthesized by nonribosomal peptide synthesis complexes [3,4]. It is known that CER has ionophoretic activities and acts as a potassium transporter. The toxic effect of CER on mammalian cells has been shown to be a result of the disturbance of the ionic equilibrium and mitochondrial transmembrane potential, which may influence the whole organism and, eventually, even cause death [5,6,7]. In foodborne outbreaks, the main clinical manifestation of CER intoxication is emesis. Therefore, CER is referred to as an emetic toxin of *B. cereus* in contrast to the group of *B. cereus* diarrheal enterotoxins (hemolysin BL, non-hemolytic enterotoxin, cytotoxin K and some others less prevalent and putative toxins).

In general, CER is produced by less than 10% of random foodborne *Bacillus cereus* isolates and is mostly related to farinaceous foods [8,9,10]. In the study of Delbrassinne *et al.* [11], CER was found in 7.4% of randomly collected rice dishes from restaurants. The prevalence increased to 12.9% in samples subjected to temperature abuse during the storage. The CER concentrations found in samples were approximately 4 ng/g of food [11]. The prevalence of emetic *B. cereus* determined in 56,899 stool samples from sporadic food poisoning cases in Korea revealed that emetic *B. cereus* was present in 0.012% of food poisoning cases [12]. An analysis of samples originating from patients suffering from diagnosed emetic food poisoning revealed CER in high concentrations in gastric fluid (4 ng/mL), blood serum (4 ng/mL), urine (8 ng/mL) and, especially, stool (160–800 ng/g) [13].

The acute effects of gastroenteritis may be easily identified with a large number of documented food poisonings as a consequence [14,15]. However, chronic effects often result from the ingestion of low to moderate levels of toxins and can be difficult to recognize. These toxin doses do not cause immediately visible symptoms, but may have a profound effect on different health aspects [16]. Quantification and characterization of these effects using biological systems could provide the information necessary for appropriate prevention and early intervention in human health protection [17].

Because the gut is the first exposure site of CER to the human body, it is highly relevant to understand the effect of sub-emetic CER concentrations on the intestinal epithelium. Colorectal cancer cell lines are often used as *in vitro* models in intestinal permeability studies [18] and the investigation of the transport characteristics of food compounds and xenobiotics. Caco-2 cells spontaneously differentiate into enterocyte-like cells upon confluency [19], which results in a polarity of the cell in an apical and baso-lateral part, separated by tight junctions. On the apical part, a brush border with microvilli is developed, which produce specific digestive hydrolases, and transport proteins, enzyme receptors, ion channels and lipid molecules are also situated on the apical part [18,20,21]. Until now, a number of toxicity studies had been performed with undifferentiated Caco-2 cells, although they did not display the characteristics of enterocytes. Moreover, differentiated tumor cells resemble normal cells and tend to grow and spread at a slower rate than undifferentiated or poorly differentiated tumor cells [22,23]. The difference in the toxicological responses of undifferentiated and differentiated cell cultures have been reported, also for Caco-2 cells [24], and some of the expressed intestinal functions of fully differentiated colon cancer cell lines, cell subpopulations and clones important for enteric pathogenesis have been described [25]. Basic *in vitro* studies of CER toxicity have been performed using different cell lines and methods, including human HeLa, Caco-2, Calu-3, Paju cells, Hep2 and natural killer cells, as well as, boar sperm cells, porcine pancreatic islets of Langerhans [26,27,28,29], most often looking into the threshold concentration of CER provoking vacuolation effects and visible mitochondrial damage. Therefore, the objective of the current study was to establish the effect of sub-emetic concentrations of CER on differentiated Caco-2 cells using assays describing the toxicity, permeability, metabolism and proteomic profile. Such a multilateral approach comprising also the investigation of long time intestinal exposure will complement current knowledge on CER toxicity.

## 2. Results and Discussion

### 2.1. Effect of Sub-Emetic Cereulide Concentrations on the Toxicity, Protein Content and Cell Morphology of Caco-2 Cells

**Figure 1:**
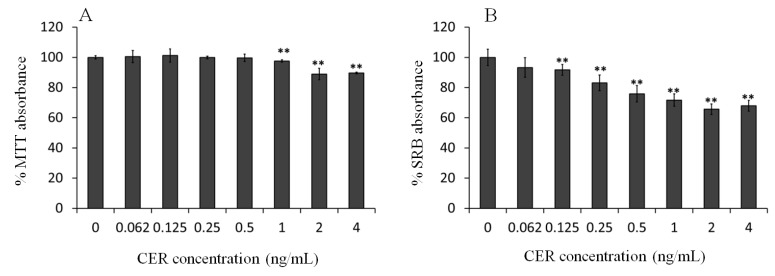
(**A**) MTT and (**B**) SRB (sulforhodamine B) results for differentiated Caco-2 cells after three-day exposure to low concentrations of cereulide (CER). Error bars indicate standard deviations between six wells. Significantly different results are indicated as: ** *p* < 0.01.

In order to determine the limit of CER toxicity, well-established assays for mitochondrial activity (MTT) and changes in protein content (SRB (sulforhodamine B)) of differentiated Caco-2 cells were evaluated after an extended, three-day exposure to low concentrations of CER (Figure 1A,B).

The MTT assay showed toxicity on differentiated cells at 1 ng/mL, whereas the SRB test already showed toxicity from a concentration of 0.125 ng/mL. In general, the SRB test appeared to be more sensitive than the MTT test to detect changes in the biological activity of differentiated Caco-2 cells. These findings are in agreement with the data of Keepers *et al.* [30], who have shown that the SRB assay provided a better linearity with cell number and a higher sensitivity on human colorectal cell line HT-29. The potential shortcomings of the MTT assay as an indicator of cellular toxicity have been reported by several authors [30,31].

**Figure 2:**
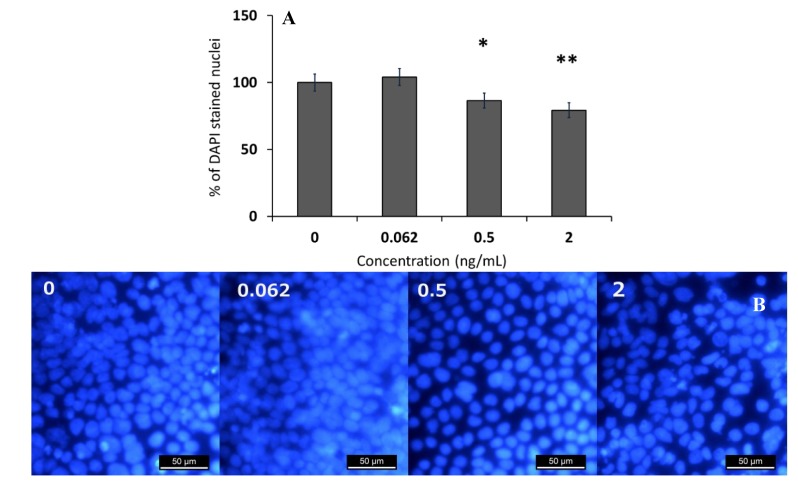
(**A**) Percentage of DAPI-stained nuclei of differentiated Caco-2 cells treated with CER (0, 0.062, 0.5 and 2 ng/mL) compared to the untreated condition; and (**B**) pictures of the DAPI-stained nuclei as observed by fluorescence microscopy. Significantly different results are indicated as: * *p* < 0.05, ** *p* < 0.01. Error bars indicate standard errors based on three wells at three technical repeats (positions) in the same well.

To elaborate on the results of the SRB reaction, an effect of CER on viable cell counts was investigated. For this purpose, the DAPI-stained nuclei of a differentiated Caco-2 cell monolayer treated with 0, 0.062, 0.5 and 2 ng/mL of CER for three days were enumerated. The results presented in Figure 2 show that CER at a concentration of 0.5 ng/mL was able to decrease the amount of adherent Caco-2 cells. In addition, the medium that was collected from differentiated Caco-2 cells treated with CER (0, 0.25 and 0.5 ng/mL) did not show any significant differences in protein content measured by the Bradford method or by quantification of the bands (stained with Coomassie blue) after 1D gel electrophoresis on a 12% polyacrylamide gel (Quantity One) (data not shown). The decreased SRB results were due to the decrease in cell number and probably not to differences in the amount of secreted proteins.

### 2.2. Effect of Sub-Emetic Cereulide Concentrations on Acidification and Lactate Formation by Caco-2 Cells

**Figure 3:**
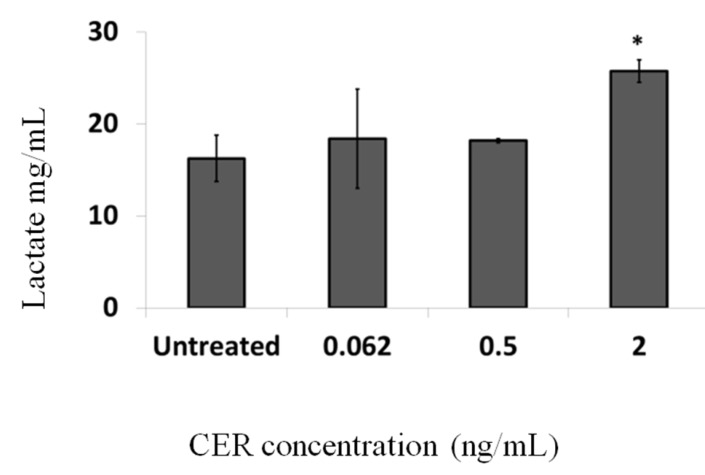
Lactate content of the cell medium after three days of treatment with 0, 0.062, 0.5 and 2 ng/mL of CER. Error bars indicate standard deviations between three wells and two technical repeats (extractions). Significantly different results are indicated as: * *p* < 0.05.

A strong difference in the color of the cell culture medium was observed after three days of treatment, with color changes starting to becoming apparent at a concentration of 0.5 ng/mL and becoming clearly visible at 2 ng/mL. The stronger acidification of the medium with higher concentrations of CER may be attributed to the increased lactate concentrations in the samples (Figure 3). The correlation between lactate profile and oxidative metabolism was suggested earlier using Chinese hamster ovary (CHO) cells, pointing to a central role of mitochondria on lactate metabolism [32]. An increase in lactate production by cells might indicate a mitochondrial dysfunction that leads to a compensatory increase in the glycolytic rate.

### 2.3. Comparison of the Mitochondrial Effects of Sub-Emetic Cereulide Concentrations between the Caco-2 MTT Assay and the Boar Sperm Test

The motility of boar semen cells has been reported as a very sensitive and convenient bioassay to determine the presence of CER (and related) toxins [27,33,34,35]. In the present study, we compared briefly the sensitivity of Caco-2 cells and boar semen cells, and the lowest concentration of CER that had a toxic effect on boar semen was 2.5 ng/mL (data not shown), whereas the effects on differentiated Caco-2 cells occurred for 1 ng/mL for the MTT test and 0.125 ng for the SRB test. If we compare these results with other sensitive techniques, such as LC-MS, in which the detection limit is 0.5 ng/gram [36], the lowest toxic dose of CER for Caco-2 cells is a factor of four lower. Therefore, we concluded that the MTT and SRB test with differentiated Caco-2 cells are at least as interesting as the boar sperm test to detect CER, but the longer incubation time also makes it more sensitive towards the effects of other matrix compounds. In the work of Jaaskelainen *et al.* [28], one of the few studies that included differentiated Caco-2 cells, the lowest CER concentration that produced visible effects on a differentiated Caco-2 cell monolayer was 2 ng/mL CER, and that in a suspension Caco-2 culture was 10–20 ng of CER/mL, in comparison to 0.125 ng/mL obtained in the current study for differentiated cells. Concentrations causing toxic effects described in our study are also lower than presumed emetic concentrations, but flank lower levels of amounts reported in the food [11,28,37,38], which indicates the high relevance of the investigations of (extended) exposure to low doses of CER.

### 2.4. Differences in Protein Expression between Treated and Untreated Caco-2 Cells

**Figure 4:**
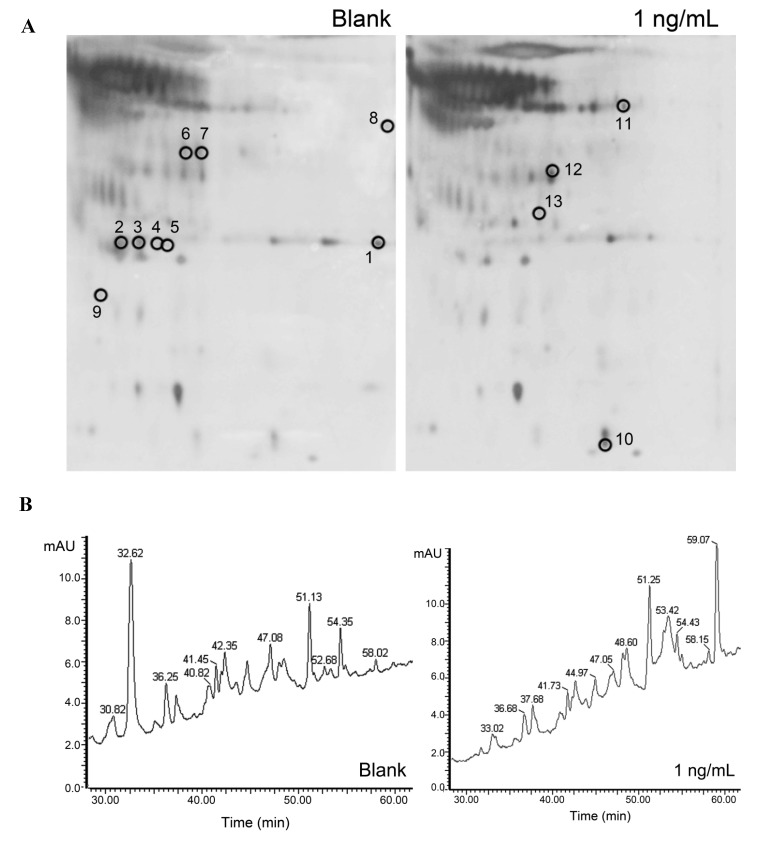
(**A**) 2D gel electrophoresis of the secreted protein fraction isolated from the cell culture medium of differentiated Caco-2 cells treated with 0 and 1 ng/mL CER for 3 days. Black circles represent protein spots that are differentially expressed. Protein spots are identified after mass spectrometry analyses and results are summarized in Table 1; (**B**) Protein profile of the cytosolic protein fraction of the same Caco-2 cells.

Table 1 and Figure 4 show the proteins that are differentially expressed by untreated and treated (CER concentration of 1 ng/mL) differentiated Caco-2 cells. Figure 4A shows the differences in protein profile based on 2D gel electrophoresis, whereas Figure 4B shows the LC profile of both untreated and treated Caco-2 cells, in which differences in peak intensities can be observed mainly in the region between 30 and 40 min and between 50 and 60 min.

Data about the effect of CER or structurally similar toxins on the expression of these specific proteins in any cell type is almost non-existent. In certain cases, data about other types of microbial and environmental toxins are available, although only a few publications have characterized them in Caco-2 cells. Most of the altered proteins correspond with defense mechanisms of the cell towards toxic compounds, including TPI1, ApoA1, PGK1, cathepsin D preprotein, LDH, GST, Prdx and Rho-GDI.

As far as we know, no literature is available about the impact of microbial toxins on IGFN, domain-containing protein 1, proprotein convertase subtilisin/kexin type 9, tyrosine 3-monooxygenase/tryptophan 5-monooxygenase activation protein, malate dehydrogenase, peroxiredoxin, copine-1 and albumin. In general, CER led towards a decrease in energy managing proteins, such as proteins involved in carbohydrate metabolism [39], TPI1, PGK1 and MDH. Triosephosphate isomerase 1 is an enzyme involved in glycolysis. It is downregulated after treatment of hepatic cell lines with certain antimicrobial peptides (a role characteristic of CER, too [40]), resulting in apoptosis [41], and with deoxynivalenol [42], which is an emetic mycotoxin from *Fusarium* species [43], with known toxicity on gut and brain cells [44]. It has also been demonstrated that neurotoxin-like beta-carbolines, which are inhibitors of mitochondrial respiration, bind to this protein [45]. Phosphoglycerate kinase is an enzyme involved in the conversion of pyruvate to lactate and plays an important role in the multidrug resistance response when overexpressed in the human ovarian cancer cell line, SW626TR [46]. Downregulation of this enzyme was observed in mouse macrophages treated with another *Bacillus* toxins, namely anthrax lethal toxin [47].

Our results demonstrated the decreased presence of apolipoprotein A-I, proapolipoprotein and apolipoprotein preprotein in the cell culture medium after CER treatment, which may be due to decreased HDL secretion. Secreted HDL is directly linked to mitochondrial respiration, more specifically, HDL increases the mitochondrial respiration and glycolysis rate by skeletal muscle cells in mice [48], and mitochondrial dysfunction through membrane potential loss of nigericin- and oligomycin-treated mice macrophages results in decreased HDL secretion [49]. In addition, the toxicity sequestering effect of HDL has been demonstrated for bacterial lipopolysaccharides [50,51,52], lipoteichoic acid [53], amphotericin [54], carcinogens [55] and *E. coli* supernatant [56]. PCSK9 was upregulated after CER treatment, and although the impact of this modified expression is currently unknown, we assume that it may participate in the altered cholesterol metabolism after CER exposure [57].

**Table 1:** Overview of the proteins that are differentially expressed between untreated and CER (1 ng/mL)-treated differentiated Caco-2 cells after three days of CER treatment. The change indicates the increase or decrease of the protein from the CER-treated cells compared to the untreated cells. M = medium, C = cytosol, N = membrane/nuclear protein fraction.

No. ^a^	Fraction	Protein	gi number ^b^	MW	pI ^c^	Coverage %	MASCOT score	Expression	Function
1	M/C	Triosephosphate isomerase 1 (TPI1)	17389815	26,625	6.5	38	145 *	-	Gluconeogenesis, glycolysis, pentose shunt
2	M	Apolipoprotein A-I (ApoA-I)	90108664	28,062	5.3	48	197 *	-	Lipid metabolism, major component of HDL, clear cholesterol from tissues, positive effect on cardiovascular diseases
3	M	Apolipoprotein A-I (ApoA-I)	90108664	28,062	5.3	56	234 *	-	Lipid metabolism, major component of HDL, clear cholesterol from tissues, positive effect on cardiovascular diseases
4	M	Proapolipoprotein	178775	28,944	5.5	39	133 *	-	Lipid metabolism, major component of HDL, clear cholesterol from tissues, positive effect on cardiovascular diseases
5	M	Apolipoprotein A-I preproprotein	4557321	30,745	5.6	37	169 *	-	Lipid metabolism, major component of HDL, clear cholesterol from tissues, positive effect on cardiovascular diseases
6	M	Immunoglobulin-like and fibronectin type III (IGFN)	257196151	383,568	5.7	32	71 *	-	Cell adhesion (wound healing), differentiation, migration, cytokine and tyrosine kinase receptors
7	M	Domain-containing protein 1	193783098	349,013	5.6	30	101 *	-	unknown
8	M	Phosphoglycerate kinase 1 (PGK1)	48145549	44,574	8.3	25	121 *	-	Conversion pyruvate to lactate, cellular response to extracellular stimulus
9	M	Nucleolar protein 5A (56kDa with KKE/D repeat), isoform CRA_b	119630990	47,898	9.4	20	74 *	-	Ribosomal action
10	M	Proprotein convertase subtilisin/kexin type 9 (Pcsk9)	149243243	74,239	6.1	41	218 *	+	Cholesterol homeostasis, LDL decrease => hypercholesterolemia
11	M	Cathepsin D preproprotein	4503143	44,524	6.1	28	118 *	+	Protease, ECM remodeling, CVD, apoptosis, immune response, tumor biomarker
12	M/C	Lactate dehydrogenase (isoform CRA_a) (LDH)	49259209/	36,516	5.9	25	110 *	+	Conversion of lactate to pyruvate, marker for cell death (marker of membrane integrity)
13	M	Deoxyribonuclease I splicing isomer 1 (DNASE1)	109809705	31,386	4.6	28	115 *	+	Waste-management endonuclease, DNA fragmentation, apoptosis
14	C	Tyrosine 3-monooxygenase/tryptophan 5-monooxygenase activation protein (YWHA)	23114	28,083	4.8	8	98 **	-	Signal transduction, trafficking, apoptosis, stress response, and malignant transformation
15	C	Malate dehydrogenase (MDH), cytoplasmic isoform 3	1255604	36,426	6.9	6	83 **	-	Citric acid cycle, gluconeogenesis
16	C	Peroxiredoxin (Prdx)	287641	22,111	8.3	9	102 **	-	Detoxification of H_2_O_2_
17	C	Glutathione S-transferase P (GSTP)	31946	23,356	5.4	13	154 **	-	Detoxification, oxidative stress
18	C	U2 small nuclear RNA auxiliary factor 2 isoform b	37545	53,501	9.2	6	74 **	-	mRNA processing
19	C	Copine-1 isoform a (CPNE1)	1791257	59,059	5.5	5	76 **	+	Membrane trafficking
20	C	Glutathione S-transferase (GST)	121730	25,631	8.9	6	81 **	+	Detoxification, oxidative stress
21	C	Rho GDP dissociation inhibitor alpha (Rho GDI 1)	36038	23,193	5.0	7	79 **	+	Activation of the oxygen superoxide-generating NADPH oxidase
22	N	Albumin, CRA p	119626079	69,348	5.92	21	89 *	+	Unknown

Notes: ^a^ Protein number. Protein numbers 1–13 (isolated from medium and separated by 2-DE) correspond spot numbers marked on 2-DE gels, Figure 2A; ^b^ GenInfo identifier in NCBInr database; ^c^ Isoelectric point; * MASCOT protein score >69 is statistically significant, based on the MASCOT scoring algorithm using a threshold of *p* < 0.5; ** MASCOT protein score >46 is statistically significant, based on the MASCOT scoring algorithm using a threshold of *p* < 0.5.

CER treatment led towards a decrease in H_2_O_2_ detoxification proteins, Prdx and GSTP [58], and to an increase in Rho GDI protein, which regulates Rho family GTPase activities. As far as we know, no data are available about decreased Prdx levels after toxin treatment. Glutathione S-transferases (GST) comprise a family of enzymes that are critical for the inactivation of toxins and carcinogens after conjugation, such as the hepatoxin microcystin LR [59] and aflatoxin B1 in rat liver tissue [60]. Incubation of HT-29 cells with alternariol also resulted in increased GST expression and activity [61]. Rho GDP dissociation inhibitor alpha controls Rho proteins homeostasis, which is involved in a wide variety of cellular functions, such as cell polarity, vesicular trafficking, the cell cycle and transcriptomal dynamics. Treatment of HepG2 with the contaminant di(2-ethylhexyl) phthalate and benzo-a-pyrene resulted in the upregulation of the Rho GDP dissociation inhibitor [62,63]. In addition, toxins from *Clostridium difficile*, *Clostridium sordellii* and *Clostridium novyi* inactivate proteins from the Rho family that regulate actin polymerization [64,65], leading towards changes in cell barrier permeability and disruption of intercellular junctions.

Cereulide exposure resulted also in elevated expression of cathepsin D preprotein. Extracellular release of pro-cathepsin D after exposure of Hela cells to *Helicobacter pylori* vacuolating toxin was demonstrated by Satin *et al.* [66]. This was the result of the incomplete conversion of the pro-cathepsin D to cathepsin D. Extracellular release of cathepsin D and the impact on cellular respiration was also observed after the addition of petroleum hydrocarbons [67]. This mechanism may also explain why cathepsin D levels were increased after valinomycin treatment, which is a microbial depsipeptide highly similar to CER [68]. In many cases, the cleavage of toxins by cathepsin D may increase their toxicity, such as for *Pseudomonas* exotoxin A [69]. Increased expression of cathepsin D was also found after LPS stimulation of microglia [70]. These results indicate the possible role of cathepsin D in apoptosis regulation after toxin treatment. The assessment of this finding should be performed, as cathepsin D is a lysosomal protease that was described to have both anti-apoptotic and pro-apoptotic functions, depending on the cause of apoptosis and probably on the applied concentration of the cytotoxic agent.

It is interesting to note that some of the proteins, differentially expressed by differentiated Caco-2 cells after CER treatment, are also biomarkers for more cancer cell phenotypes. Increased cathepsin D activity is observed in carcinomas compared to in normal tissues and may play a role in tumor growth and metastasis [71,72]. GSTs are enzymes considered as dedifferentiation markers [73] and are also upregulated in breast cancer tissue and in the distal colon, which is the site with the highest incidence of colorectal cancer [74,75]. Furthermore, Rho-GDI has been upregulated in hepatic cancer cells [76]. Based on these results, we may carefully consider a possible role of sub-emetic levels of CER and maybe other toxins in the development of cancer. The observed increase in lactate production may contribute to the previous note, as mitochondrial oxidative stress in cancer cells drives lactate production [77]. These findings urgently require further exploration.

## 3. Experimental Section

### 3.1. Cell Culture

Caco-2 (ATCC HTB37) were cultivated in Dulbecco’s Modified Eagle’s Medium (DMEM) with Glutamax (Gibco, Langley, OK, USA), supplemented with 10% fetal bovine serum (FBS, Greiner Bio-One, Wemmel, Belgium), 1% non-essential amino acids, 2% penicillin/streptomycin and transferrin (Invitrogen, Merelbeke, Belgium) at 10% CO_2_ in air. The culture medium of the cells was changed every other day. Caco-2 cells with a confluency degree of approximately 80% were subcultured to maintain their undifferentiated character and, hence, the rapid growth of the cells. During the subculturing in a T25 flask, cells were first washed with 4 mL phosphate saline buffer (PBS) without calcium and magnesium, and then, 2 mL trypsin were added to the flask. After 20 s of rinsing the cells, the overload of trypsin was removed until 1 droplet was still in the flask, and the cells were incubated at 37 °C for 5 min. Finally, the cells were suspended in 5 mL of medium and distributed in the new T25 flask at a splitting ratio of 1 to 5. Caco-2 cells were grown to 95% confluency in T25 culture flasks for continuous subcultivation of the cells, whereas differentiated Caco-2 cells were obtained by extended culturing of 21 days, as reported earlier [78,79]. The cell morphology was analyzed by phase-contrast microscopy (Leica DMI3000 B, Leica, Solms, Germany) equipped with a camera (Leica DFC420C, Leica, Solms, Germany).

### 3.2. Cereulide (CER)

Cereulide, kindly provided by Dr. Paul in’t Veld (Netherlands Food and Safety Authority), was originally synthesized from tert-butyl carbonate (Boc)-D-Ala-OH, H-L-O-Val-OBn (where Bn is a benzyl moiety), Boc-L-Val-OH and H-L-O-Leu-OBn by Chiralix B.V., (Nijmegen, The Netherlands), as described elsewhere [80]. The working stock of 50 ng/µL was used to make appropriate working dilutions in dimethylsulfoxide (DMSO, Sigma-Aldrich, St. Louis, MO, USA) in the range of 0–2 ng/mL.

### 3.3. Toxicity Tests

The effect of CER on Caco-2 cells was characterized with two established assays. The MTT assay ((3-(4,5-dimethylthiazol-2-yl)-2,5-diphenyltetrazolium bromide) was used to measure the mitochondrial activity, as described by the protocol of Mosmann [81]. The SRB assay (sulforhodamine B) was used for the measurement of cellular protein content, according to Vichai and Kirtikara [82]. For the toxicity assay, cells were trypsinized, counted according to the Trypan blue staining method with a Bürker counting chamber, and seeded at a concentration of 40,000 cells per well in a 96-well plate. To determine CER toxicity on undifferentiated Caco-2 cells, the treatment was applied one day after seeding the cells, whereas for differentiated cells, the treatment was applied after 21 days. MTT and SRB reactions were measured one and three days after the CER treatment. Six biological repeats were made for each CER concentration in each of the MTT and SRB toxicity tests. The cells were treated with CER for three days to mimic an extended exposure time.

An additional toxicity evaluation was done using a computer-aided boar semen bio-assay in which the effect of tested concentrations of CER on boar semen motility was evaluated. The assay was performed as described previously [1,34], with the modification that DMSO was used as a negative control and that the exposure time was 20 min. The indication of acute toxicity was a complete cease of semen motility within the 20 min of exposure. The blank sample (semen exposed to DMSO) served as an exposure control (no cease in motility for 20 min). The assay was performed in duplicate.

To complement the SRB results and see whether CER treatment affected the cell number, Caco-2 cells were seeded on glass coverslips in 24-well plates at a concentration of 100,000 cells per well, allowed to differentiate for 21 days and then exposed to 0, 0.062, 0.5 and 2 ng/mL of CER in serum-free medium. After the exposure, the cell nuclei were stained with 4′,6′-diamino-2-phenylindole (DAPI) at a concentration of 1 μg/mL. Per biological repeat (3 per concentration), the amount of cells per camera snapshot of the monolayer at 3 random places in the well (upper left, middle and lower right part) were counted. Images of stained cells were obtained with a Leica DMR microscope equipped with a fluorescence imaging system (100× magnification).

### 3.4. Lactate Analysis

Lactate extraction was performed by mixing 1 mL of cell culture medium with 4.5 mL of water and 0.5 mL of an internal standard solution (phenyl-α-D-glucopyranoside, 0.8 mg/mL water). The solution was shaken for 15 minutes and filtered (nitrogen free filter, 595 ½, diameter of 185 mm, SCHLEICHER & SCHUELL, Dassel, Germany). A volume of 500 µL was retained; 10 µL of a 3M NaOH solution were added, and the sample was dried under nitrogen. The dry residue was derivatized to trimethylsilyl esters using 400 µL of N,O-bis(trimethylsilyl) trifluoroacetamide, 50 µL of trimethylchlorosilane and 500 µL of pyridine. Analyses were carried out using a Varian 3380 gas chromatograph equipped with a flame-ionization detector (Varian Instrument Group, Walnut Creek, CA, USA). The chromatographic parameters were: stationary phase, (5%-phenyl)-methylpolysiloxane; film thickness, 0.25 µm; 30 m × 0.32 mm inside diameter (i.d.) (Agilent Technologies, Palo Alto, CA, USA); mobile phase, He at 1 mL/min, split 1/40; injector temperature, 250 °C; detector temperature, 340 °C; injection volume, 1 µL; column temperature program, 60 °C for 5 min, ramp at 10 °C/min to 290 °C, then isothermic for 10 min. The flame ionization detector was operated with hydrogen and air at respectively 30 and 300 mL/min and helium at 20 mL/min as makeup gas.

### 3.5. Preparation of Protein Fractions for Proteomics

For the proteomics experiment, cells were seeded in 24-well plates at a concentration of 100,000 cells per well and allowed to differentiate for 21 days. Prior to CER treatment (0 and 2 µg/mL in serum-free medium), cells were washed trice with serum-free medium.

#### 3.5.1. Secreted Protein Fraction

After CER exposure, the Caco-2-containing culture medium of the T25 culture flask was centrifuged (1000× *g*, 10 min), and the supernatant was transferred through a 0.22-µm sterile filter (Millipore). The medium was then concentrated by ultrafiltration (10 kD filter; Millipore, 40 min, 2000× *g*), until approximately 250 µL of retentate were obtained. Small variations in the final volume were adjusted to an equal volume by adding sterile PBS. Secreted proteins were precipitated after the addition of an aqueous 7.5% trichloroacetic acid (TCA) solution (w/v), as described by Chevallet *et al.* [83]. After protein precipitation, the pellet was dissolved in 0.35 mL of isoelectric focusing (IEF) buffer. The buffer was prepared by dissolving 7 M urea, 2 M thiourea, 4% CHAPS in water (w/v).

#### 3.5.2. Cytosolic Protein Fraction

Caco-2 cells were washed and treated with 1 mL of mild lysis buffer, containing 1% Triton X-100 and 1% NP-40 in Ca^2+^/Mg^2+^ supplemented PBS (v/v), as well as protease inhibitors (2 M phenylmethanesulfonyl fluoride), 10 mg/L leupeptin, 10 mg/L apoprotinin, 1 mM NaVO_3_, 2.5 g/L Na_4_P_2_O_7_ and 0.1 mM NaF). The lysate was centrifuged (10 min, 14,000× *g*), and the supernatant was retained as the cytosolic protein fraction.

#### 3.5.3. Nuclear/Membrane-Bound Protein Fraction

The pellet obtained after lysate centrifugation of the above-described procedure was dissolved in 250 µL Laemmli 1× lysis solution (0.125 M Tris-HCl,123 g/L glycerol, and 23 g/L SDS), sonicated for 10 s and centrifuged (14,000× *g*, 10 min).

### 3.6. 1D and 2D Gel Electrophoresis

The secreted protein fraction was separated by 2-DE [84]. In the first dimension, the linear immobilized pH gradient strips (17 cm, pH 3–10) were used, and IEF was carried out with a Bio-Rad Protean IEF Cell (Hemel Hempstead, UK). The second dimension, SDS-PAGE electrophoresis was run through 1 mm-thick 12% polyacrylamide gel. The proteins were visualized by silver staining.

Differential display analysis of 2-DE gel data sets was obtained by comparing images of three control gels and three gels of CER-treated samples. Master gels were used to obtain the differences between protein profiles of control and CER-treated cells. Densitometry analysis was performed with image analysis software (Discovery Series PDQuest 2-DE analysis software package version 7.4.0 [85]) integrated into a VersaDoc 4000 Imaging System (Bio-Rad, Hemel Hempstead, UK).

The nuclear/membrane-bound protein fraction was separated by 1D SDS-PAGE, to avoid coating the charge of the proteins by negatively-charged SDS, through 12% polyacrylamide gel and then visualized by Coomassie blue staining [86]. Three control and three CER-treated gels were used.

All reagents used for protein preparations were of electrophoresis grade and were purchased from Bio-Rad (Hertfordshire, UK), and all protein preparations were stored at −20 °C until analysis.

#### In-Gel Digestion

All visible spots obtained after Coomassie staining (1-DE gels) and differentially displayed protein spots obtained after silver staining (2-DE gels) were excised and subjected to tryptic in-gel digestion in accordance with the procedure described elsewhere [87]. After digestion, the extracted peptides were purified with C_4_ ZipTip columns (Millipore, Darmstadt, Germany) and evaporated to dryness in SpeedVac (Eppendorf, Hamburg, Germany). Dried samples were mixed with α-cyano-4-hydroxycinnamic acid (CHCA) 1:5, v/v (5 mg/mL; Waters, Milford, MA, USA) and spotted onto a metal MALDI plate.

### 3.7. Cap-LC

Cytosolic protein fractions were purified by Sep-Pak Vac 1cc Diol Cartridges using a vacuum manifold solid phase extraction system (Waters, Milford, MA, USA). Diol cartridges were conditioned in three steps: firstly by aspirating and dispensing to waste three times 0.5 mL of 80% acetonitrile (ACN) mixed with 20% water solution of 0.1% trifluoroacetic acid (TFA) (v/v), secondly by 50% ACN mixed with 50% water solution of 0.1% TFA (v/v) and, finally, by an aqueous solution of 0.1% TFA (v/v). The protein sample solution was loaded onto the cartridge and washed five times with 0.5 mL aqueous solution of 0.1% TFA (v/v). Proteins were eluted from the column with 0.35 mL of 80% ACN mixed with 20% aqueous solution of 0.1% TFA (v/v). The eluting solution was evaporated to dryness in a SpeedVac (Eppendorf, Germany), then proteins were reconstituted in 0.1 mL of 25 mM NH_4_HCO_3_ (pH 7.3) and subjected to tryptic digestion (final concentration of 20 µg/mL) for 18 h at 37 °C.

The CapLC system (Waters, Milford, MA, USA) equipped with a UV/VIS detector coupled to Tempo™ LC MALDI spotter (Applied Biosystems, Foster City, CA, USA) was used for peptide separation and collection directly onto the MALDI plate. Chromatographic separation was performed on a silica based Symmetry 300™ C_18_ column (300 µm × 150 µm I.D., 3.5-μm particle size, Waters, Milford, MA, USA) at 30 °C. The flow rate was 2 µL/min, and the injection volume was 10 μL. Vial temperature was maintained at 5 °C in the autosampler tray. Mobile phase A consisted of 0.1% TFA aqueous solution and mobile phase B consisted of 80% ACN mixed with 20% aqueous solution of 0.1% TFA (v/v). Eluted peptides were detected by UV absorbance at 280 nm. The 100-min gradient elution was programmed to increase over 70 min with Solvent B from 5% to 80% and then to condition the column back to the initial conditions until the completion of the run. The spotter make-up flow was set to 2 μL/min (5 mg CHCA matrix dissolved in 1 mL of 50% ACN aqueous solution).

Duplicate injections were acquired for three CER-treated and three control samples.

### 3.8. Mass Spectrometry

Mass spectrometry acquisition (MS) was performed with a MALDI TOF/TOF 4800 Plus analyzer (Applied Biosystems, Foster City, CA, USA) equipped with a 200-Hz, 355-nm Nd:YAG laser. Acquisitions were performed in positive ion reflectron mode. The instrument parameters were set using the 4000 Series Explorer software, version 3.5.3 (Applied Biosystems Inc., Foster City, CA, USA). Mass spectra were obtained by averaging 1800 laser shots covering a mass range *m/z* 800–4000. Internal calibration and external calibration of the mass range were performed with tryptic autolysis fragments for in-gel and LC samples, respectively. After the recording of the MS spectra of the secreted protein fraction (2-DE gels), ten of the most intensive precursor signals were selected for MS/MS analysis. MS/MS analysis was achieved under 1 kV collision energy in positive ion mode with air used as a collision gas. The differential display analysis performed in the nuclear/membrane-bound protein fraction (1-DE gel) was based on a comparison of peptide ion masses between control and treated samples (presence/absence) in MS spectra according to the protein migration position in the gel. A cytosolic protein fraction differential display (Cap-LC fractions) was performed in accordance with the peptide retention time and spot position obtained after chromatographic elution. MS/MS spectra were achieved only for peptide ions that were found as differentially expressed (the limit was 10 S/N in MS spectrum).

### 3.9. Protein Identification

Protein identification and database searching was performed by GPS Explorer Software v3.6 (Applied Biosystems Inc., Foster City, CA, USA). A combined ion search using MS and MS/MS data were matched against the NCBInr by MASCOT search engine [88]. The parameters were set as follows: two missed trypsin cleavages and oxidation of methionine with 21 ppm mass tolerance.

### 3.10. Statistical Analysis

Mean values and corresponding standard deviations and coefficients of variance of six-times-repeated SRB and MTT reactions were computed for each test condition. The statistical significance of the observed differences between tested conditions were analyzed using IBM SPSS Statistics 20 (Chicago, IL, USA). In the first step, the normality of the data were investigated using the Kolmogorov–Smirnov test. To determine whether the data were significantly different (*p* < 0.05, indicated with *, and *p* < 0.01, indicated with **), a *t*-test (two-tailed with unequal variance) was applied.

## 4. Conclusions

While acute doses of CER are found in foods and cause a large number of documented food poisonings, the remaining question of scientific and public health relevance is the prevalence and the effect of sub(acute)-doses [17]. These CER doses do not cause immediately visible symptoms, but may have profound effects on different health aspects. The results of current study support this hypothesis, and show that the lowest toxic dose of CER for non-differentiated Caco-2 cells is 0.062 ng/mL and 0.125 ng/mL for differentiated Caco-2 cells. The impact on differentiated cells, a more relevant epithelium model, was noticeable using different toxicity markers and the proteomic profile of treated cells.
